# Case Report: The Role of Neuropsychological Assessment and Imaging Biomarkers in the Early Diagnosis of Lewy Body Dementia in a Patient With Major Depression and Prolonged Alcohol and Benzodiazepine Dependence

**DOI:** 10.3389/fpsyt.2020.00684

**Published:** 2020-07-15

**Authors:** Caroline Bouter, Niels Hansen, Charles Timäus, Jens Wiltfang, Claudia Lange

**Affiliations:** ^1^Department of Nuclear Medicine, University Medical Center Göttingen (UMG), Georg-August-University, Goettingen, Germany; ^2^Department of Psychiatry and Psychotherapy, University Medical Center Göttingen (UMG), Georg-August-University, Goettingen, Germany; ^3^German Center for Neurodegenerative Diseases (DZNE), Goettingen, Germany; ^4^Neurosciences and Signaling Group, Department of Medical Sciences, Institute of Biomedicine (iBiMED), University of Aveiro, Aveiro, Portugal

**Keywords:** dementia with Lewy bodies, 123I-FP-CIT, 18F-FDG-PET, dementia, biomarker

## Abstract

Dementia with Lewy bodies (DLB) is the second most common form of dementia and is assumed to be often under- or misdiagnosed, especially in early stages. Here we present a complex case of probable DLB with major depression and alcohol and benzodiazepine dependence in which DLB was ruled out initially. This case highlights the challenging diagnostic workup of DLB patients. Core clinical features can be missing and indicative biomarkers can be negative, especially in early stages of the disease. Initially, Fluorodeoxyglucose positron emission tomography as well as neuropsychological assessment were suspicious for a possible DLB diagnosis in our patient while core clinical criteria were missing and the indicative biomarker 123I-FP-CIT SPECT was negative. Follow up was performed two years later and the patients showed several core and supportive clinical features of DLB and 123I-FP-CIT SPECT showed a pathological pattern. Extensive neuropsychological assessment in combination with PET imaging might provide crucial evidence for DLB even in early stages. If neuropsychology and PET imaging point to an early DLB diagnosis careful follow-up should be performed as core symptoms and indicative biomarkers might appear in later stages of the disease.

## Introduction

Dementia with Lewy Bodies (DLB) is the second most common form of dementia following Alzheimer’s disease (AD) (1). DLB is clinically characterized by dementia with fluctuating cognition with the addition of deficits in the extrapyramidal motor system, hallucinations, or other psychiatric symptoms as well as REM sleep behavior disorder or autonomous dysfunctions with syncope and falls (2). It is assumed that DLB is often under- or misdiagnosed as core symptoms might be absent especially in early stages or symptoms might overlap with Alzheimer’s or Parkinson’s disease (3).

Clinical diagnosis of DLB includes the presence of several core symptoms or the combination of core symptoms with indicative biomarkers (**Table 1**). For the diagnosis of a probable DLB at least two core clinical features or a combination of one core clinical feature and one indicative biomarker have to be present. A possible DLB can be diagnosed if either one core clinical feature or one indicative biomarker is present. Imaging biomarkers include 123I-FP-CIT SPECT, 123I-MIBG scintigraphy, Fluorodeoxyglucose positron emission tomography (18F-FDG-PET) and Magnetic resonance imaging (MRI). While 123I-FP-CIT SPECT and 123I-MIBG myocardial scintigraphy are classified as indicative biomarkers in the clinical criteria for DLB, 18F-FDG-PET, and MRI are listed as supportive biomarkers (4).

**Table 1:** Criteria for the clinical diagnosis of Dementia with Lewy bodies (DLB).

Criteria for the clinical diagnosis of DLB	Index patient	
	2017	2019
**Dementia**	+	+
**Core clinical features**		
Fluctuating cognition in attention and alertness	–	–
Visual hallucinations	Under low dose amitriptyline	+
Parkinsonism	–	Bradydiadochokinesis, Tremor
REM sleep behavior disorder	–	–
**Supportive clinical features**		
Severe sensitivity to antipsychotic substances	–	–
Postural instability	–	+
Repeated falls, syncope or other transient episodes of unresponsiveness	–	–
Severe autonomic dysfunction	–	–
Other hallucinations	–	acoustic
Systematizes delusions	–	–
Apathy, anxiety, depression	+	+
**Indicative biomarkers**		
Abnormal 123I-FP-CIT SPECT	–	+
Abnormal 123I-MIBG myocardial scintigraphy	na	na
Polysomnographic confirmation of REM sleep without atonia	na	na
**Supportive biomarkers**		
Relative preservation of medial temporal lobe structures	–	na
Generalized low cortical uptake in FDG-PET or perfusion SPECT	+	na
Prominent posterior slow-wave activity in EEG	–	na

For the diagnosis of a probable DLB at least two core clinical features or a combination of one core clinical feature in combination with one indicative biomarker have to be present. A possible DLB can be diagnosed if either one core clinical feature or one indicative biomarker is present. Results of the index patient are indicated as “+” when present and “-” when absent; na, not available.

Furthermore, neuropsychological tests (especially attentional, executive and visuo-constructive tests) can provide a profile to differentiate DLB from AD, but the role of extensive neuropsychological assessment in the diagnostic workup of DLB is unclear (5, 6).

To date, therapeutic management of DLB includes a range of pharmacological and non-pharmacological strategies to modify cognitive and psychotic symptoms. Clinical guidelines recommend the use of choline esterase inhibitors as rivastigmine or donepezil. The use of antipsychotics for the acute treatment of hallucinations or behavioral disturbances is not recommended as severe neuroleptic sensitivity can occur (2).

Here, we present a complex case of probable DLB with major depression and alcohol and benzodiazepine dependence in which DLB was ruled out initially. We want to discuss the role of imaging biomarkers and neuropsychological tests in the workup of patients with suspected DLB.

## Case Report

A 63-year-old male patient was taken to the emergency room at New Year’s 2017 with alcohol and benzodiazepine intoxication. The patient reported that he suffered from alcohol dependence and has been in remission for about 10 years. This was his first relapse, but he was not able to recall the reason for it. Suicidal intoxication could not be excluded; therefore, the patient was transferred to the department of psychiatry.

When examined, the patient was in a good general condition according to his age with an obese dietary condition. Neurological and physical examination showed no pathologic findings, especially no signs of early neurological signs of DLB as bradykinesia and rigidity. The patient had a history of recurrent depressions lasting over more than 10 years leading to several hospitalizations. He underwent out-patient psychotherapy until 4 weeks earlier and was concomitantly treated with sertraline (100 mg), quetiapine (600 mg), opipramol (300 mg), agomelatine (25 mg), and lorazepam (2 mg). He reported being highly dependent on the help of his wife in every day activates and the patient’s wife confirmed that he was showing progressive cognitive deficits at home and also reported about recent changes in his personality and behavior. She reported that her husband shows an obsessional behavior, especially in his day’s schedule. Even minor changes were not tolerated and led to furious behavior.

**Figure 1:**
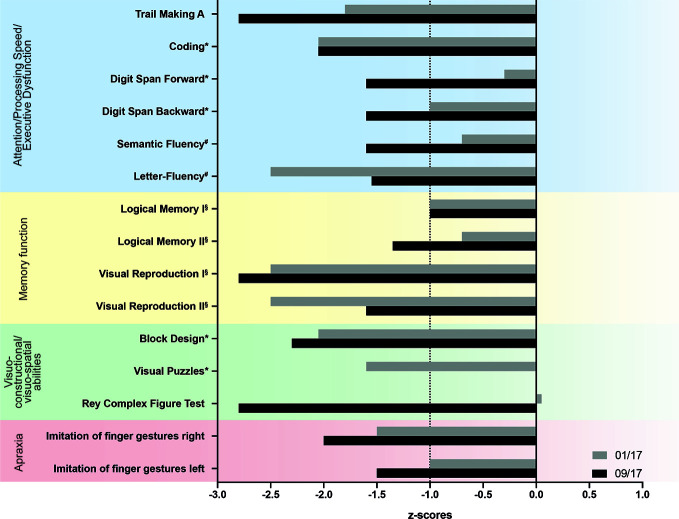
Results of neuropsychological tests. The patient was examined thoroughly twice in 2017. Results are listed as z-scores in relation to normal results. The dashed line indicates the cut off for pathological scores adjusted for age. Trail Making B was also performed, but the patient was not able to finish the test. Furthermore, the patient was not able to complete the visual puzzles test in the follow up examination in 09/17. Tests included: *Wechsler Adult Intelligence Scale – Fourth Edition; ^§^Wechsler Memory Scale – Fourth Edition and ^#^Regensburger verbal fluency test.

During the in-patient stay, the patient showed deficits in memory function, orientation, and concentration. Therefore, he was further examined neuropsychologically. Extensive neuropsychological tests showed deficits in visuoconstruction, mental rotation, psychomotor and processing speed as well as cognitive flexibility, visual memory, letter fluency, and ideomotor apraxia fulfilling criteria for dementia. Diagnosis of dementia was made according to the 5^th^ edition of the Diagnostic and Statistical Manual of Mental Disorders (DSM V), criteria A – D were fulfilled. Overall, test results, especially the parietal dysfunction, pointed to an early neurodegenerative process consistent with a possible early-onset Alzheimer’s disease (EOAD) or Lewy Body dementia (**Figure 1**).

**Figure 2:**
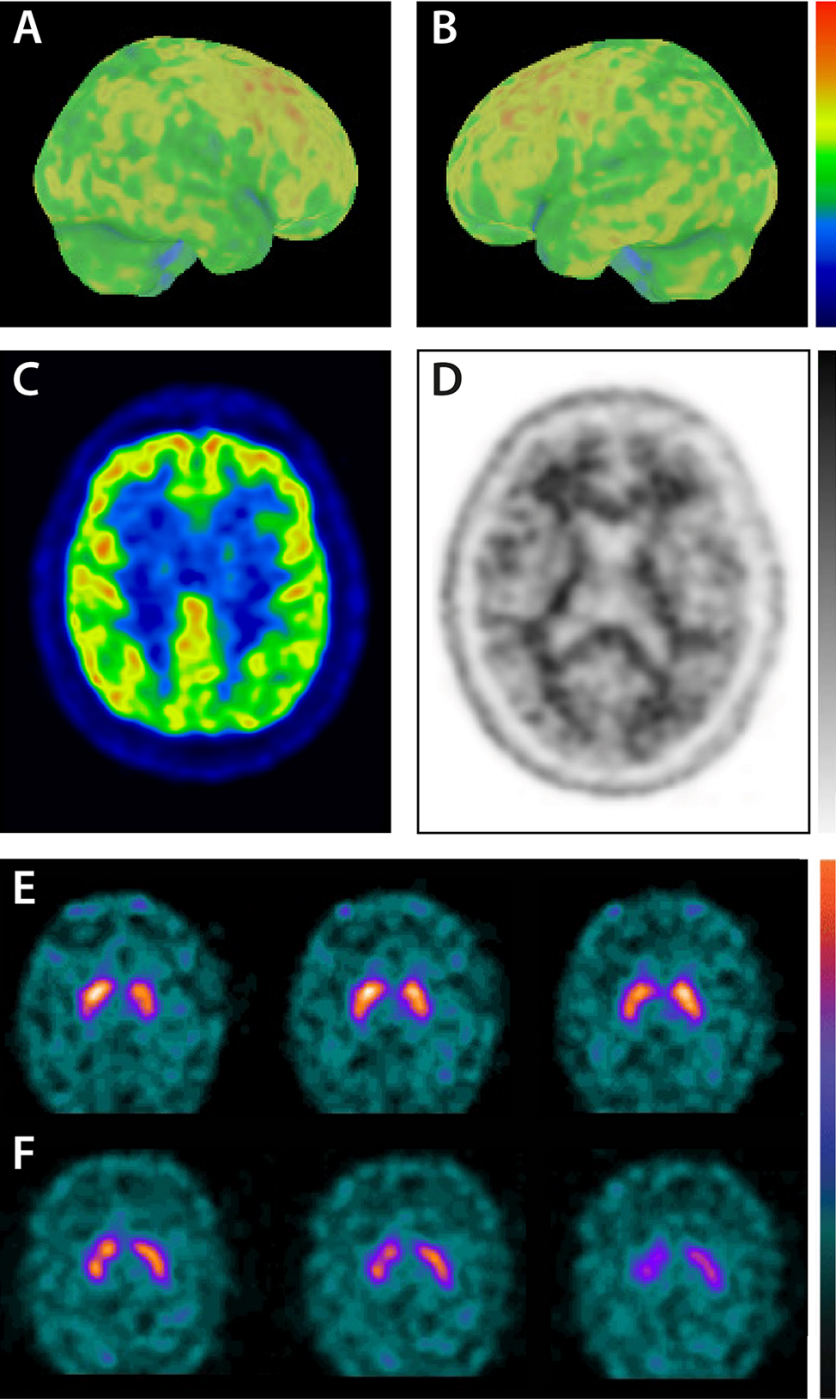
Imaging biomarkers. **(A–C)** 18F-FDG-PET results. Three-dimensional surface projections **(A, B)** show distinct bilateral temporo-parietal and occipital hypometabolism. **(A)** Right hemisphere; lateral view. **(B)** Left hemisphere; lateral view. **(C)** Hypometabolism is relatively spared in the posterior cingulate gyrus compared to occipital areas (cingulate island sign); transverse view. **(D)** 18F-Florbetaben-PET did not show increased amyloid burden. **(E**, **F)** Dopamine transporter imaging with 123I-FP-CIT SPECT in transverse slices. While the initial scan was negative **(E)**, follow up showed lower uptake within the right putamen **(F)**.

Further neuroimaging was performed. While MRI did not show any pathologies, 18F-FDG-PET showed bilateral hypometabolism of occipital, parietal and temporal lobe, as well as frontal lobes to a lesser extend with relative sparing of the posterior cingulate gyrus (cingulate island sign; **Figures 2A–C**). Amyloid PET with 18F-Florbetaben was negative (**Figure 2D**). Dopamine transporter imaging with 123I-FP-CIT SPECT was further done and binding ratios (left and right putamen and caudate nucleus in reference to occipital cortex) were calculated. However, it did not show a nigrostriatal deficit (binding ratios: right caudate nucleus 2.37; left caudate nucleus 2.39; right putamen 2.44; left putamen 2.39; **Figure 2E**).

Analysis of cerebrospinal fluid biomarkers showed elevated levels of total Tau (953 pg/ml; reference >450 pg/ml) while phosphor-181-Tau, amyloid-β42, and amyloid-β40 were within normal range. Further diagnostics included EEG, which was normal. No sleep disturbances were described by the patient.

The patient was treated for depression with sertraline (200 mg), quetiapine (350 mg), and pipamperone (120 mg). Benzodiazepine withdrawal was done. He was released in a stable condition without lorazepam medication three months after admission to the hospital. AD was ruled out as a possible diagnosis due to amyloid PET results. The diagnosis of DLB could not be established at this point either. As the etiology of the cognitive impairment remained unclear, follow-up was scheduled four months later.

In July 2017, the patient presented with a relapse of benzodiazepine abuse and major depressions at the department of psychiatry. He was admitted as an in-patient and benzodiazepine withdrawal was initiated. Neuropsychological tests were repeated for follow-up and showed progressive deficits indicating dementia. Test results still pointed to an EOAD or DLB. During the in-patient stay, the patient also showed visual hallucinations after a low-dose treatment with amitriptyline, saying he could see birds flying out of the wall. However, DLB was still not a probable diagnosis according to the criteria for the clinical diagnosis of DLB (**Table 1**) even though neuropsychological tests showed frontoparietal dysfunction suspicious for DLB. Medication with sertraline was replaced by venlafaxine (225 mg) and risperidone (1.5 mg) was added for the treatment of psychotic symptoms. The medication was well-tolerated, especially with no extrapyramidal side effects that appeared during the 5-months long hospitalization.

Two years after the initial presentation, the patient was seen for another follow-up in January 2019. He was still using alcohol and benzodiazepines irregularly and showed recurring depressive episodes. Furthermore, he reported about acoustic hallucinations in the form of dialogical voices. He also described recurrent visual hallucinations in the form of emerging animals which were well-formed and detailed fulfilling the criteria for visual hallucinations according to (7). Treatment included venlafaxine (225 mg), quetiapine (400 mg), and risperidone (4 mg).

**Figure 3:**
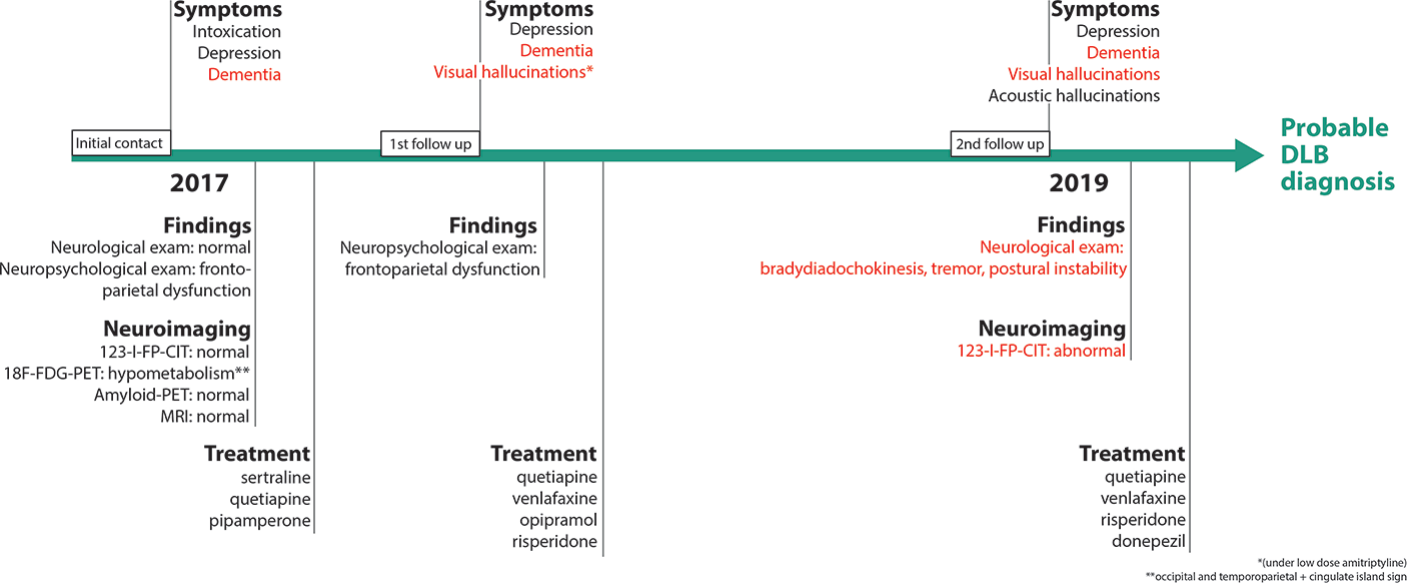
Timeline of symptoms, clinical findings, imaging biomarker, and treatment. Criteria for the clinical diagnosis of Dementia with Lewy bodies are indicated in red.

Neurological examination showed bilateral bradydiadochokinesis, tremor, and postural instability. Due to the new core and supportive clinical features, 123I-FP-CIT SPECT was repeated. 123I-FP-CIT SPECT showed lower uptake mainly within the right putamen with lower binding ratios in both putamina and left caudate nucleus compared to 2017 (binding ratios: right caudate nucleus 2.42; left caudate nucleus 1.94; right putamen 1.83; left putamen 1.91; **Figure 2F**). In accordance with clinical findings and 123I-FP-CIT SPECT results, the clinical diagnosis of probable DLB was made. The patient was given donepezil (10mg) and discharged from the hospital in April 2019 after cognitive and psychotic symptoms stabilized. Cognitive functions remained unchanged until the last follow-up visit in May 2019. **Figure 3** summarizes symptoms, clinical findings, imaging results, and treatment.

## Discussion

Our patient was initially hospitalized as a case of major depression with alcohol and benzodiazepine abuse and acute intoxication with possible suicidal intentions. Due to his appearance during the in-patient stay and his cognitive history provided by his wife, a possible dementia was suspected and further diagnostics were initiated.

As the patient was initially hospitalized due to intoxication, the diagnosis of dementia might have been easily missed. Furthermore, the diagnosis of DLB remained unclear as core clinical features for DLB were missing and the indicative biomarker 123I-FP-CIT SPECT was negative at first. Due to several key factors, a DLB diagnosis could be established: (1) Careful anamnesis, especially information provided by the patient’s wife, was taken and a possible neurodegenerative disorder was considered. (2) An extensive neuropsychological assessment was performed. The patient’s neuropsychological profile showed dementia according to DSM V. Profiles matched EOAD or DLB profiles. (3) Molecular imaging affirmed a possible DLB diagnosis with 18F-FDG-PET and amyloid PET. 18F-FDG-PET showed a typical pattern of DLB including the cingulate island sign while amyloid PET showed a normal pattern, excluding AD as a possible diagnosis. (4) Follow-up finally confirmed the diagnosis of probable DLB as core symptoms appeared and 123I-FP-CIT SPECT as an indicative biomarker was positive two years after the initial hospitalization.

Diagnosis of DLB is very challenging, especially in the early stages of the disease as the clinical presentation of DLB is very variable. Diagnosis relies on clinical criteria provided by the Consortium on DLB which were recently revised in 2017 (2). However, sensitivity of the clinical diagnosis of DLB is only about 20% in early stages of the disease (3). The diagnosis of DLB is further complicated by the fact that the prodomal phase of dementia with Lewy bodies includes (1) mild cognitive impairment (MCI), (2) delirium onset, and (3) psychiatric-onset presentations (7).

While in our case presented here, core clinical features and indicative biomarkers according to the diagnostic criteria of DLB were missing in 2017, neuropsychological assessment and PET imaging pointed to the diagnosis of DLB.

Global reduced cortical 18F-FDG-PET is listed in the diagnostic criteria for DLB as a supportive biomarker. Especially in early stages of the disease 18F-FDG-PET can be an important biomarker as it detects changes in cerebral glucose metabolism even before neurodegeneration. In DLB cases, 18F-FDG-PET typically shows hypometabolism in occipital (especially primary visual cortex) and temporoparietal cortex areas with a relatively preserved posterior cingulate cortex (cingulate island sign; **Figure 2C**). The cingulate island sign shows high specificity differentiating AD and DLB (8). However, so far 18F-FDG-PET is only used as a supportive biomarker in LDB diagnosis due to several reasons. While initial studies with a limited number of patients showed a high correlation of hypometabolism in the primary visual cortex with histopathological findings distinguishing DLB from AD, larger studies showed insufficient sensitivity and specificity (9–11). Furthermore, interpretation of 18F-FDG-PET can be difficult as patterns of lower FDG uptake can be very similar to AD patients. Therefore, 18F-FDG-PET might not be able to prevent misdiagnosis of DLB.

To distinguish between AD and DLB, imaging biomarker 123I-FP-CIT SPECT is more suitable showing high sensitivity and specificity (12). 123I-FP-CIT binds with high affinity to presynaptic dopamine receptors detecting dopaminergic nigrostriatal degeneration which is common in DLB. Reduced striatal uptake of 123I-FP-CIT highly correlated with a clinical diagnosis of DLB showing a very good diagnostic accuracy for distinguishing DLB from AD in pathologically confirmed DLB (12, 13). Furthermore, a combination of amyloid PET and 123I-FP-CIT SPECT showed high accuracy in the differentiation between different forms of dementia (14, 15).

However, a normal 123I-FP-CIT SPECT does not exclude DLB with minimal brainstem involvement. Some DLB patients do not develop parkinsonism showing intact dopaminergic neurons in the substantia nigra and therefore a true negative 123I-FP-CIT SPECT (16). Lewy body pathology is assumed to affect the substantia nigra in an early stage of the disease followed by amygdalae, limbic cortex, and neocortex (17). However, some DLB cases without involvement of the lower brain stem have been reported (18–20). Therefore, Lewy body pathology might start in neocortical areas in some patients leading to a negative 123I-FP-CIT SPECT. Furthermore, a recent study by van der Zande et al. reported that 123I-FP-CIT SPECT can initially be rated as normal while patients show abnormal scans in follow up (21). Findings in our case are in line with these results showing a positive scan in follow-up after the initial negative scan supporting the hypothesis of a neocortical subtype of DLB disease progression from rostral to caudal. Follow-up imaging of the nigrostriatal system might provide valuable information to avoid misdiagnosis, especially in complex cases as the one presented here.

Furthermore, neuropsychological tests played an important role in the diagnostic workup of our case revealing a major neurocognitive disorder. So far, the role of neuropsychological assessment in the diagnosis of DLB remains unclear even though several tests show high accuracy in the differentiation of AD from DLB (2, 4, 5, 22).

Several studies showed that deficits in attentional function were more severe in DLB patients compared to AD patients (23–26). DLB patients show a greater impairment on visuospatial and constructional tasks compared to AD patients while DLB patients show better results in episodic memory function than AD patients (27–31). Our patient showed deficits in visuospatial and constructional tasks, a significant reduced psychomotor processing speed, and an apraxia for imitation of figure gestures matching this profile.

Tests on attention, visuoperceptual and spatial functions and apraxia seem to be a valuable tool for the diagnosis of DLB and neuropsychological assessment covering the full range of cortical domains affected by DLB should be included.

The described patient showed deficits in frontoparietal functions that are also seen in AD patients. EOAD was initially considered as another possible diagnosis due to neuropsychological findings. Patients with EOAD have often a similar profile with a predominant non-amnestic syndrome with deficits in visuospatial abilities, praxis, or other non-memory cognition (32, 33). Furthermore, our patient had visual memory deficits which could be a hint for AD, but since verbal memory was not or only mildly impaired this is not a typical finding for AD. However, amyloid PET was negative excluding EOAD as a possible diagnosis in this case as the method shows a very high negative predictive value.

Even though neuropsychological assessment is an objective testing of different brain functions a possible influence of alcohol and drug abuse, as seen in our patient, should be considered in diagnostics. It could be shown that alcohol dependence impairs cognitive functions including memory and executive functions, speed of processing, verbal fluency as well as attention, and visuospatial functions (34, 35).

Alcohol dependence is a known risk factor for the development of dementia. It is suggested that neurotoxic effects of ethanol and acetaldehyde lead to structural and functional brain damage. Chronic alcohol consumption can lead to cognitive impairment due to brain lesions mainly including executive functions, episodic memory, and visuospatial capacities (36, 37). Furthermore, prolonged alcohol dependence is associated with the Wernicke-Korsakoff syndrome due to thiamine deficiency. However, it is unclear which cerebral areas are affected the most by alcohol dependence. While some studies describe deficits mainly in frontal lobe functions, others describe right hemisphere functions as more susceptible to effects of alcoholism, and other studies demonstrated diffuse brain deficits (38). Equalities of those findings to the described cognitive profile and 18F-FDG-PET results of our patient are not described. Another important factor for the diagnosis of DLB is the ideomotor apraxia seen in our patient. Imitation of finger gestures is often impaired in LDB patients and not known to be influenced by alcohol or benzodiazepine abuse (22).

Overall, a combination of extensive neuropsychological tests and imaging biomarkers FDG-and amyloid PET supported the diagnosis of DLB even before core clinical features occurred.

DLB can be easily misdiagnosed, especially if core symptoms and indicative biomarkers are absent. Furthermore, the prodromal phase of the disease including MCI, delirium-onset, or psychiatric-onset presentations displays a diagnostic challenge (7). Both neuropsychological tests covering the full range of cortical domains affected by DLB and PET imaging should be considered as valuable tools in the early diagnostic workup of DLB to help to establish a diagnosis of a possible LDB with the urgent need of follow-up to provide early diagnosis and early treatment of the disease. In the described case, treatment included antipsychotic drugs. Quetiapine was used to treat symptoms of depression. Risperidone was added for augmentation at the first follow up. Due to progredient psychotic symptoms, the treatment with risperidone was continued before the diagnosis of DLB was established, especially before a nigrostriatal deficit has been shown in 123I-FP-CIT SPECT. The use of risperidone in DLB can lead to neuroleptic malignant syndrome and delirium in about 50% of cases (39–42). However, a potential benefit of atypical antipsychotics in the treatment of psychoses associated with dementia is also described. A small number of studies were able to show the efficacy of risperidone or the combination of risperidone and levodopa in the treatment of DLB (40, 42–44). While severe sensitivity to antipsychotic substances is commonly seen in DLB cases (and therefore listed as supportive clinical feature in the diagnosis of DLB), missing sensitivity to risperidone does not exclude DLB. However, risperidone has to be used very carefully in dementia patients starting with low doses as risperidone might not be well tolerated. Furthermore, possible drug-induced parkinsonism had to be considered in our case after starting risperidone and quetiapine treatment. However, extrapyramidal side effects usually appear shortly after starting risperidone medication which was not the case in our patient (45). A recent systematic review on the role of quetiapine in patients with parkinsonism showed no effect of quetiapine on motor symptoms (46). And 123I-FP-CIT SPECT results excluded drug-induced parkinsonism in follow up.

Furthermore, medication might also influence cognitive deficits. However, if standard dosage is used no side effects on cognitive function are expected (47). Quetiapine was intermittently used in a higher dosage of 600 mg/d that could have worsened cognitive function to a minor extent. Risperidone was used in a lower dosage possibly not affecting cognition. Massive visual-spatial deficits and an apraxia for finger gestures as seen in our patient cannot be explained by the medication. These findings cannot be caused neither by medication nor by substance abuse.

The impact of supporting biomarkers of DLB should also be considered. The patient presented here showed very early neuropsychiatric symptoms as depressive symptoms have been present many years before the manifestation of cognitive symptoms followed by parkinsonism. Little is known about the onset and progression of DLB symptoms. However, the incidence of depression in dementia is around 30%–40% of cases (48, 49). Major depression might be the first sign of dementia and is described as a risk factor for the development of dementia (50, 51). In DLB prevalence of depressions is assumed to be higher compared to AD and therefore depressions are listed as supportive clinical feature in the criteria for clinical diagnosis of DLB (2). However, identification of patients with psychiatric symptoms that convert to DLB remains difficult as no formal criteria for psychiatric-onset DLB are available, especially due to the limited number of studies so far (7).

Diagnostic steps should be performed carefully in the clinical routine as DLB should not be ruled out easily if core symptoms are missing or indicative biomarkers are negative. Supportive clinical features, supportive biomarkers or neuropsychology might point to a DLB diagnosis in early stages and follow-up should be performed continuously. However, the impact of supportive features remains unclear and should be studied further.

## Conclusion

Especially in early stages of the disease core clinical features of DLB can be missing and indicative biomarkers can be negative. Therefore, detailed neuropsychological tests in combination with PET imaging might provide crucial evidence for DLB even in early stages. If neuropsychology and PET imaging point to an early DLB diagnosis, as seen in the described case, careful follow-up should be performed as core symptoms and indicative biomarkers might appear in later stages of the disease.

## Data Availability Statement

The datasets generated for this study are available on request to the corresponding author.

## Ethics Statement

Written informed consent was obtained from the patient for the publication of any potentially identifiable images or data included in this article.

## Author Contributions

CB wrote the manuscript and participated in study design. NH, CT, and JW participated in the discussion of results. CL designed the project. All authors contributed to the article and approved the submitted version.

## Funding

This work is supported by the Open access publication fund of the University Medical Center Göttingen.

## Conflict of Interest

The authors declare that the research was conducted in the absence of any commercial or financial relationships that could be construed as a potential conflict of interest.
